# The Most rapid recurrent meningioma: Case report and literature review

**DOI:** 10.1016/j.ijscr.2023.107939

**Published:** 2023-02-18

**Authors:** Novan Krisno Adji, Komang Yunita Wiryaning Putri, Laksmi Indreswari, Muhammad Yuda Nugraha, Ali Habibi

**Affiliations:** a Department of Neurosurgery, Soebandi Regional Hospital, Jember, Indonesia; b Department of Neurology, Soebandi Regional Hospital, Jember, Indonesia; c Department of Sergery, Soebandi Regional Hospital, Jember, Indonesia; d Faculty of Medicine, University of Jember, Indonesia

**Keywords:** Anaplastic, Brain tumor, Case report, Meningioma, Recurrence, Surgical

## Abstract

**Introduction:**

Meningioma, a primary intracranial neoplasm, accounts for 36 % of all primary brain tumors. Approximately 90 % of cases are benign. Malignant, atypical and anaplastic meningioma potentially have more recurrence risk. In this paper, we report a rapid recurrence of meningioma that is probably the most rapid recurrence for either benign or malignant type.

**Case presentation:**

This paper reports a case of rapid meningioma recurrence 38 days after the first surgical resection. The histopathological examination showed suspicion of anaplastic meningioma (WHO grade III). The patient has a history of breast cancer. After total surgical resection, there was no recurrence reported until three months, and the patient was planned for radiotherapy. Only several cases have been reported about the recurrence of meningioma. With recurrence the prognosis is poor, and two patients died several days after treatment. The primary treatment for the entire tumor was surgical resection, and several issues were combined with radiotherapy. In this case, the recurrence time from the first surgery was 38 days. The most rapid recurrent meningioma reported to this day was 43 days.

**Conclusion:**

This case report showed the most rapid onset of recurrent meningioma. Therefore this study cannot show reasons for the rapid onset of recurrence.

## Highlights

• Rapid recurrence of meningioma in 38 days after first surgical resection.
• The most rapid onset of recurrence in meningioma
• Malignant meningioma
• Possibility due to breast cancer history of patient

## 1 Introduction

Meningioma is one of the major subgroups of intracranial neoplasms that account for around 36 % of all primary brain tumors [1]. Approximately 90 % of meningioma cases are benign. According to the WHO classifications, grade I meningioma is the most common subtype, whereas grades II and III are designated for atypical and anaplastic neoplasms, respectively [2]. Surgical resection is a significant and effective treatment for meningioma. The atypical and anaplastic meningioma are potentially more recurrence risk (29–52 % and 50–94 %, respectively) than type I meningioma (∼10 %) after complete resection [3]. This paper reports a case of rapid meningioma recurrence in one month. We did not find any examination that indicates it was a malignant or benign type of meningioma. Based on the summary of the literature review that we have seen, this case probably has the most rapid recurrence time for either benign or malignant meningioma. This case report has been reported in line with the SCARE Guideline Criteria 2020 [4].

## 2 Case presentation

A 54-year-old woman was brought to the emergency department complaining of weakness in the left arm and leg for four days and persistent headaches for one month. The headache was getting worse for the past week with nausea without vomiting. The patient also complained there was a lump on the right side of the head since three months ago, which was increasing. Based on the historical anamnesis, the patient has a history of left breast cancer and had a tumor resection one year ago.

**Fig. 1:**
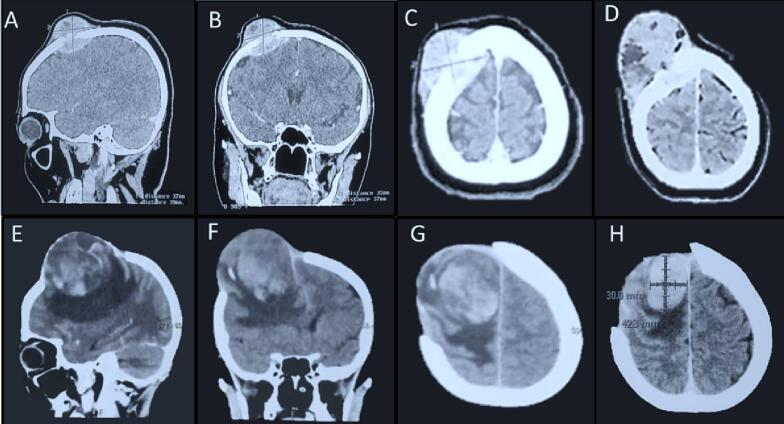
A–C. First head CT with contrast, there was a solid mass, D. Evaluation of head CT after two months, E-F. Head CT examination one month after surgical resection, H. The progression of head CT evaluation after one week.

Based on physical examination, the patient is fully-conscious (GCS E4-M6-V5), and there was a left hemiparesis. There was a lump in the right frontal; the patient had been examined with head computed tomography (CT) with contrast two months before. Based on the first head CT with distinction, there was a solid mass, extra-axial supratentorial, lobulated edges, measuring 3.8 × 3.1 × 3.2 cm in the convexity of the right frontal bone, and there was substantial heterogeneous contrast enhancement (Fig. 1A–C). The patient evaluated the CT scan with contrast in the emergency department. The result showed that the mass was progressively growing with the size of 5.8 × 5.0 × 5.1 cm and pressing the right frontal lobe to the medial side, causing a midline shift of 0.9 cm (Fig. 1D).

**Fig. 2:**
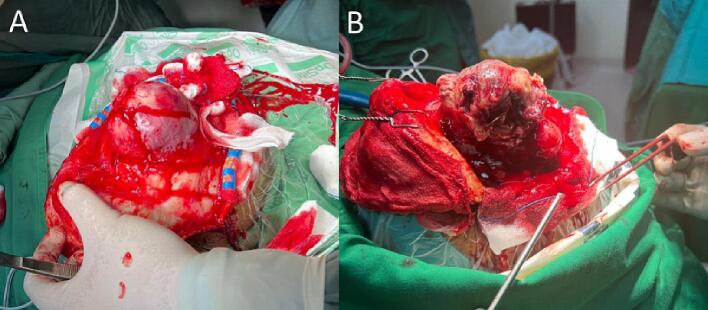
A. First surgical finding, B. Recurrence surgical finding.

A histopathological examination was not performed on the patient because it was considered possibly benign meningioma. Then the patient planned surgery for tumor excision. Right frontal craniotomy carried out total tumor resection (Fig. 2A). Then the patient was evaluated three days after surgery; based on the physical examination, the patient was fully conscious and without neurological deficit. After five days of evaluation in the hospital, the patient was fully recovered and out of the hospital. The patient did not plan additional treatment (e.g., radiotherapy).

Thirty-eight days after the first surgical resection, the patient came to the outpatient clinic due to a left hemiparesis. The patient was planned to head a CT scan examination. Head CT scan examination showed the mass was re-growing with the size of 3.0 × 4.0 cm in the exact location (Fig. 1E–F). Then the patient was planned for pre-surgical resection of the recurrence tumor. One week after the patient came to the clinic, awaiting the assignment of the surgical schedule, the patient was brought to the emergency department due to loss of consciousness (E1-M4-V1), hemiparesis, and there was anisocoria pupil. Then the head CT evaluation was performed in the emergency department and showed a result size of 3.0 × 4.2 cm, and there was focal brain edema (Fig. 1H). The patient was planned for urgent surgical resection (Fig. 2B). Then the tumor tissue was examined with histopathological examination.

**Fig. 3:**
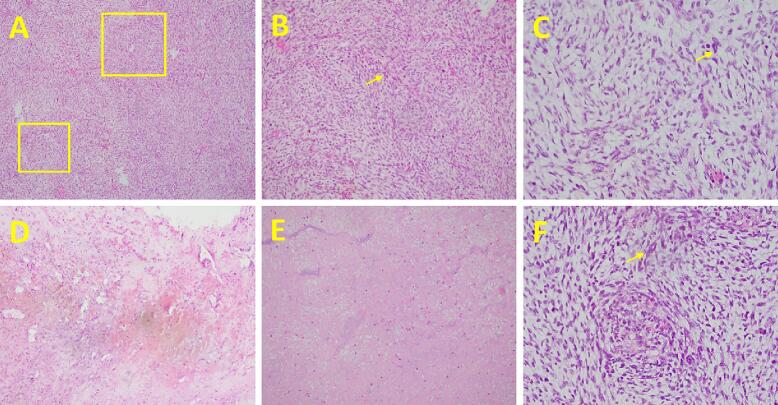
A. Whirlpool structured by spindle cell, H&E 10×, B. Atypical cell, H&E 40×, C. Moderate to severe atypical cell, H&E 100×, D. Bleeding area, H&E 4×, E. Necrotic cells spot, H&E 10×, F. Abnormal mitosis, H&E 100×.

Based on the results of histopathological examination, it was found that spindle cells with moderate to severe atypical oval nuclei formed a whirlpool with abnormal mitoses. The tumor preparations also found bleeding; some tissue had extensive necrosis (Fig. 3). Based on the results of this examination, it was concluded that the pathological diagnosis was suspicion of anaplastic meningioma (WHO grade III). The limitation of this case is not being able to determine whether the tumor cells are primary or are metastases from previous cancers due to the lack of availability of immunohistochemistry examination. The patient's outcome was good, the patient was compos mentis on five days post-surgery, but there was still slightly left hemiparesis. There was no recurrence report until three months after surgery, and the patient was planned for radiotherapy.

## 3 Discussion

Only several cases have been reported of meningioma recurrence (Table 1). Most of the tumor sites were in the frontal or temporal region. In this case report, the tumor site was in the frontal region, both primary and recurrence tumors. The main clinical manifestation was contralateral hemiparesis. Several cases also have the main clinical manifestation of hemiparesis [5]. The primary clinical manifestation mostly depends on the location of the mass.

**Table 1:** Summary of literature review about recurrent meningioma cases.

Study	Location	Clinical manifestations	Recurrence period	Radiological mass findings	Pathological findings	Treatment	Prognosis
Ashmore et al.*,* 2021 [13]	Scalp of frontoparietal	Ptosis of the right eye	13 years	4 × 4 cm firm and fixed mass	WHO grade I	Surgical excision	Indicated probable further recurrence
Baltus et al.*,* 2019 [14]	Right parietal parasagital	Generalized tonic-clonic seizures	6 months	Large tumor recurrence and in the contralateral side	WHO grade II	Subtotal resection and radiotherapy	n/a
Bujko et al.*,* 2017 [5]	Parietal convexity	Epileptic seizures and paralysis of the right lower limb	1 year, 20 months, and 1 year	1. Recurrence tumor with sagital sinus invasion 2. Recurrence tumor with sagital sinus invasion 3. Recurrence tumor with cerebral falx and occipital invasion	1. WHO grade III 2. WHO grade III 3. WHO grade III	1. Surgical resection and radiotherapy 2. Gross total resection 3. Resection	Indicated further recurrence
Jalisi*,* 2012 [7]	Frontal bone and sinus	Nasal mass, intermittent headache, and anosmia	n/a	Recurrence tumor with frontal and sinus extending through the right cribiform plate into the ethmoid sinuses to the middle meatus	WHO grade II	Gross total resection and radiotherapy	Fully recovered without deficit. No recurrence in 5 years.
Read and Williams, 2017 [15]	Cervicomedullary junction	Neck pain and rapid onset of arm and leg weakness	5 years	1,4 cm enhancing mass at cervicomedullary junction	WHO grade I	Systemic doxorubicin	n/a
Sadiya et al.*,* 2019 [16]	Left frontal	Slurred speech	1 year	2,9 × 2,5 × 2,4 cm solid cyctic tumor of left frontal dural base with invasion of brain parenchyma	WHO grade II-III	Subtotal resection and radiotherapy	n/a
Shingai et al.*,* 2021 [17]	Sphenoid	Intermittent diplopia	7 years	Tumor mass in the posterior part of sphenoid with right oculomotor invasion	WHO grade II	Resection and gamma knife radiotherapy	n/a
Thomas et al.*,* 2019 [18]	Right middle fossa	Generalized tonic-clonic seizures	7 years, 2 years, 5 years, and 2 years	2,4 × 2,9 × 1,5 cm right middle fossa with foramen ovale invasion	WHO grade II	Surgical resection, bevacizumab and everolimus	n/a
Wang et al.*,* 2015 [19]	Right orbitofrontal	Serious Headache, Gradually impaired vision of the right eye, and vomiting	3 years, 2 years, and 5 months	1. Small local reccurence tumor 2. Large tumor with frontal and temporal dural invasion 3. Larger tumor with dura, brain, and sphenoid sinus invasion	1. WHO grade II 2. WHO grade III 3. WHO grade III	1. Subtotal resection + Single-fraction gamma knife radiotherapy 2. Subtotal resection	Patient died 8 days after coma
Zahid et al.*,* 2021 [20]	Left parietal	Generalized tonic-clonic seizures	2003–2017	Multiple sites of new meningiomas and regrowth at primary tumor site	WHO grade III since 2016	8 surgical + fractioned external beam radiotherapy, gamma knife radiotherapy, everolimus + octreotide, and Lu-dotatate	Patient died after 5 months of Lu-dotatate therapy

The primary treatment for the entire tumor was treated with surgical resection (total or subtotal), and several cases had been combined with radiotherapy. In this case, we only treated the patient with complete surgical resection. The use of adjuvant radiotherapy in high-grade meningioma management remains controversial. No significant correlation was identified between postoperative radiation and the outcome of recurrent high-grade meningioma [6]. In the summary of cases (Table 1), there was only one study that reported full recovery of patients with atypical meningioma (WHO grade II) that were treated with radiotherapy [7].

Based on the summary of several cases, most have poor prognostic of recurrence, and in two cases, the patient died several days after treatment. The prognosis of anaplastic meningioma type is poor, with high recurrence rates. Overall, the survival time is less than two years, and the median time to recurrence is 9.6–42.1 months [8]. In this case, the recurrence time from the first surgery was 38 days. The most rapid recurrent meningioma reported to this day was 43 days [9].

The most rapid recurrence study [9] states that the short time to recurrence might correlate to the sub-total removal even though the authors underlined that the extent of surgery is not associated with the onset of recurrence. The possible cause, in this case, is a hormonal influence on tumor formation and progression due to the patient's historical breast cancer disease. Schoenberg et al. in 1975 [10] described, for the first time, an increased incidence of meningiomas in a group of patients diagnosed with breast cancer. In 1979 Donnell et al. [11] described the importance of estrogen receptors (ER) and progesterone receptors (PR) in meningioma cells. The absence of PR's or ER's expression is related to more aggressive behavior, higher risk of progression, and higher recurrence [12]. However, this case report cannot directly show the association between the rapid onset of recurrence with breast cancer. Therefore, it needs further study to identify the correlation.

## 4 Conclusion

This case report showed the most rapid onset of recurrence of meningioma. Therefore, this study cannot show the associated factor the rapid onset of recurrence. This case also showed that total resection could give good prognostic of patient survival in recurrent meningioma.

## Consent

Written informed consent was obtained from the patient and family.

## Ethics approval

The study includes human participants to report was approved by the ethics committee of Soebandi General Hospital.

## Funding

N/A.

## Declaration of competing interest

N/A.
